# Aortic Dissections in the Elderly: Older Age in Patients With Acute Aortic Syndromes Is Associated With Delayed Time to Surgery

**DOI:** 10.7759/cureus.70355

**Published:** 2024-09-27

**Authors:** Sorasicha Nithikasem, George Hung, Abhishek Chakraborty, Srujanesh Gunda, Seung W Baek, Hirohisa Ikegami, Gengo Sunagawa, Mark J Russo, Leonard Lee, Anthony Lemaire

**Affiliations:** 1 Surgery, Rutgers Robert Wood Johnson Medical School, New Brunswick, USA; 2 Cardiac Surgery, Rutgers Robert Wood Johnson Medical School, New Brunswick, USA; 3 Cardiothoracic Surgery, Robert Wood Johnson University Hospital, New Brunswick, USA

**Keywords:** elderly population, survi outcome, thoracic aorta, time-to-treatment, type a aortic dissection

## Abstract

Objective: In the setting of acute aortic syndromes, timely access to definitive surgical repair is of paramount importance. Older patients, primarily septuagenarians and octogenarians, undergoing emergent ascending arch repair experience higher rates of mortality compared to younger patients. Despite this risk, studies show that surgical management is still superior to medical management for this patient population. The objective of this study is to determine if older age impacts the time from presentation to the start of surgery for patients with acute aortic syndromes undergoing surgical repair.

Methods: This retrospective review included all patients with acute aortic syndromes who underwent emergent ascending aortic arch repair from January 2018 to May 2023 at a single academic institution. Our analysis compared outcomes for older patients (age 70 years and older) with younger patients (age less than 70 years). Primary outcomes included 30-day mortality, postoperative stay, time from emergency department presentation to the start of surgery, and time from diagnosis with computed tomography to the start of surgery. Secondary outcomes included postoperative complications. Outcomes were analyzed using chi-squared and Student's t-tests, with significance set at p<0.05.

Results: Of 107 patients included (male, N=57), 71 (66%) were under the age of 70 and 36 (34%) were 70 years of age or older. The younger cohort had more male and non-White patients, with no differences in rates of hypertension, dyslipidemia, and smoking history. With no difference in the rate of transfers from outside hospitals, we observed longer times from presentation to the start of surgery for older patients compared to younger patients (7 hours and 13 minutes vs. 6 hours 25 minutes; p=0.02) and also for time of diagnosis to the start of surgery (4 hours 22 minutes vs. 3 hours 54 minutes; p=0.006). Older patients had higher rates of intraoperative (0% vs. 17%, p<0.001) and 30-day (7% vs. 44%, p<0.001) mortality. There were no differences in length of stay or in rates of postoperative complications and surgery-related emergency department visits.

Conclusions:Patients aged 70 and older experienced delays from the time of presentation to the start of surgery and from time of diagnosis to the start of surgery. Age should not delay an individual from receiving timely transfer to a tertiary center for a higher level of care to better assess the patient’s operative candidacy and determine appropriate treatment.

## Introduction

In the setting of acute aortic syndromes (AAS), timely access to definitive surgical repair is of paramount importance [1]. Such acute aortic pathologies include acute aortic dissections (AADs), intramural hematomas (IMH), and penetrating aortic ulcers (PAU), all of which carry high morbidity and mortality without prompt surgical management with aortic arch repair [1,2]. The literature has established a time-dependent increase in mortality for patients with AAD, with an incremental increase of 1-2% per hour in the first 24 hours, culminating in a peak risk of mortality occurring at the 48-hour mark from symptom onset [2-5]. The proportion of patients with AADs who are managed surgically has increased to 90% over the last two decades [6]. The increase in surgical management rates, combined with improved surgical technique and imaging technologies, has been associated with a decrease in in-hospital mortality and improved outcomes for patients with AAS [2,6].

Older patients, primarily septuagenarians and octogenarians, undergoing emergent ascending aortic arch repair experience higher rates of mortality and longer hospital lengths of stay compared to younger patients [7-9]. These findings of older patients having worse outcomes can be possibly attributed to (1) poor tissue quality and (2) more comorbidities in these patient age groups. Despite this increased operative risk in older patients, some studies have suggested that surgical intervention for AAS in suitable candidates may lead to enhanced outcomes and an improved quality of life [10]. Subsequently, age alone should not be the primary determining factor in whether a patient receives surgical repair.

There is a paucity of studies investigating the potential delays to care that older patients experience in the setting of acute aortic pathologies. Very few studies have characterized the impact that age has on time of management, specifically from time of presentation at an emergency department (ED) to the time of surgery [5]. The objective of this study was to determine the impact that age has on the time from presentation to the start of surgery for patients with AAS undergoing surgical repair.

This abstract was previously presented at the AATS Aortic Symposium on April 25, 2024. The research project received Ethical approval by the Rutgers University Institutional Review Board.

## Materials and methods

Methods

This retrospective review included all patients with AAS who underwent emergent ascending aorta and/or arch repair from January 2018 to May 2023. This study was performed at a single academic institution designated as a tertiary care center for aortic syndromes. Our analysis compared outcomes between older patients (defined as those of age 70 years and older) and younger patients (defined as age less than 70 years). Primary outcomes included intraoperative mortality, 30-day mortality, and postoperative length of stay. Other primary outcomes included the time from ED presentation to the start of surgery and the time from diagnosis with computed tomography (CT) scan to the start of surgery. The time of ED presentation was defined as the time of patient arrival and immediate triage by ED personnel. The time of diagnosis with CT was defined as the time when the CT scan was read by the radiologist and the diagnosis was confirmed and communicated to the attending physician in the ED.

Secondary outcomes included postoperative complications occurring during hospitalization, which included postoperative bleeding requiring intervention, cerebrovascular accident, pericardial effusion requiring a pericardial window, and pleural effusion requiring thoracentesis. Other postoperative complications included ED visits related to the index surgery occurring within 30 days of patient discharge. Outcomes were analyzed using chi-squared tests, Fisher’s exact tests, Student’s t-tests, and Mann-Whitney U tests, with significance set at p<0.05.

The primary inclusion criteria included patients diagnosed with aortic dissections or intramural hematomas of the ascending aorta and other pathologies of the ascending aorta. The primary exclusion criteria were all patients with aortic pathology that did not involve the ascending aorta or who were treated without surgery. No patients who were considered non-operative and preferred medical management were included in the study. All surgical interventions occurred within one to two hours of diagnosis.

## Results

Of 107 patients included in this study, 71 (66%) were under the age of 70 and 36 (34%) were 70 years of age or older. The younger cohort had more males (80% (n=71) vs. 44% (n=36), p<0.001) and fewer White non-Hispanic patients (55% (n=71) vs. 75% (n=36), p=0.04). There were no differences in rates of preoperative comorbidities such as hypertension, dyslipidemia, smoking history, chronic obstructive pulmonary disease, atrial fibrillation, diabetes mellitus types I/II, drug abuse history, and Marfan syndrome (Table 1).

**Table 1:** Baseline characteristics. ^*^Significance set at p<0.05. ^**^Significance set at p<0.01. Student’s t-test was used to compare continuous variables, and chi-squared test was used to compare categorical variables, with alpha set to 0.05.

Variable	Overall (n=107)	Younger patients (age <70 years) (n=71)	Older patients (age >70 years and older) (n=36)	p-value
Baseline characteristics				
Age (years) (median, IQR)	63 (54-74)	58 (50-63)	78 (73-81)	<0.001**
Gender (male), n (%)	73 (68)	57 (80)	16 (44)	<0.001**
Race (White non-Hispanic), n (%)	66 (62)	39 (55)	27 (75)	0.040*
Body mass index (median, IQR)	27 (24-32)	27 (24-33)	28 (23-31)	0.370
Comorbidities				
Hypertension, n (%)	99 (93)	64 (90)	35 (97)	0.188
Dyslipidemia, n (%)	46 (43)	27 (38)	19 (53)	0.145
Smoking history, n (%)	41 (38)	29 (41)	12 (33)	0.450
Chronic obstructive pulmonary disease, n (%)	3 (3)	1 (1)	2 (6)	0.204
Atrial fibrillation n (%)	10 (9)	5 (7)	5 (14)	0.221
Diabetes mellitus type I/II, n (%)	8 (7)	5 (7)	3 (8)	0.764
Drug abuse history, n (%)	6 (6)	6 (8)	0 (0)	0.079
Marfan syndrome, n (%)	2 (2)	2 (3)	0 (0)	0.320

**Figure 1:**
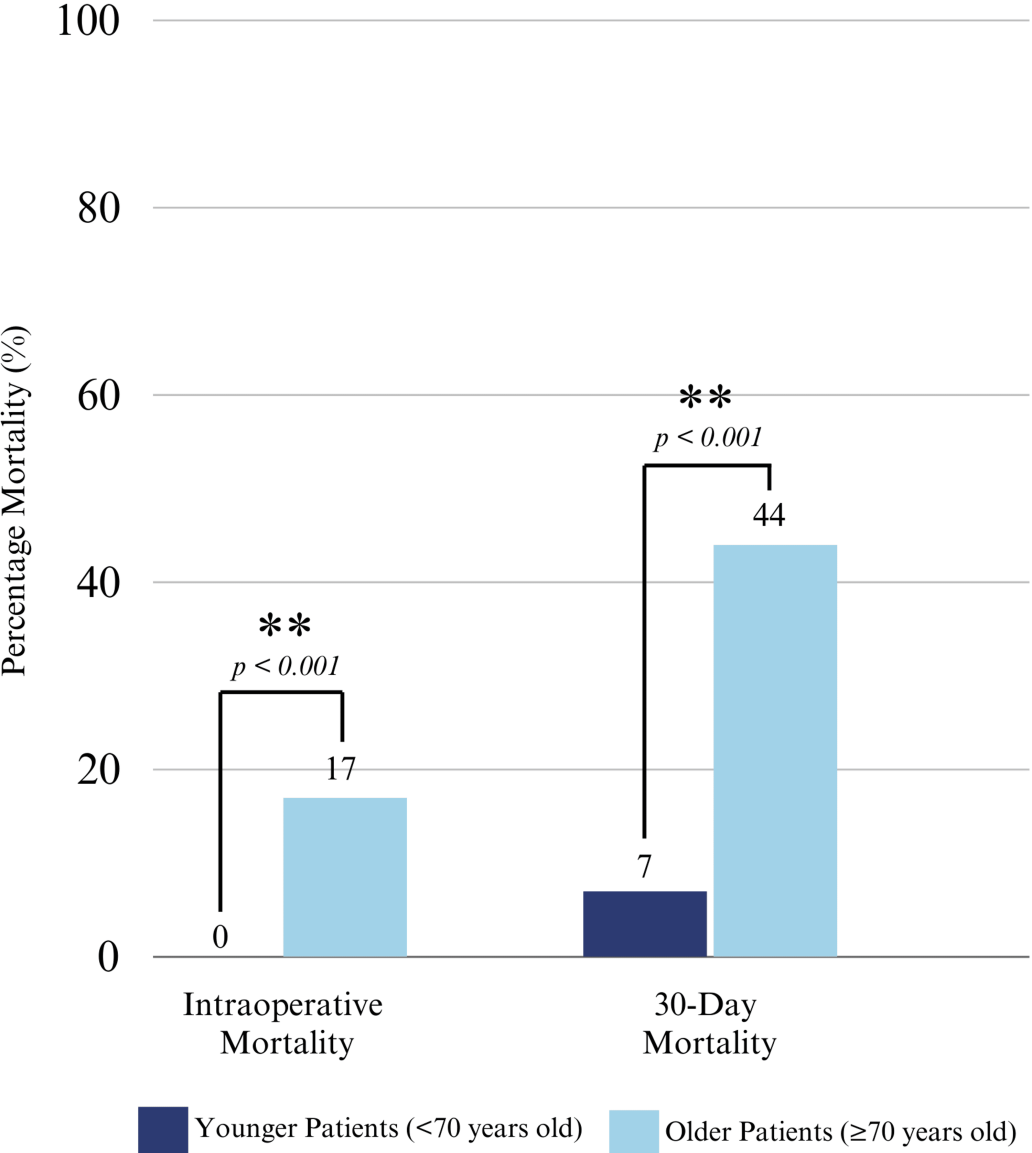
Surgical mortality.

Our analysis of the primary outcomes demonstrates that younger patients had lower rates of intraoperative mortality compared to older patients (0% (n=71) vs. 17% (n=36), p<0.001) and lower rates of 30-day mortality (7% (n=71) vs. 44% (n=36), p<0.001). There were no differences in postoperative length of stay (nine days (n=71) vs. eight days (n=36), p=0.397) (Figure 1; Table 2).

**Table 2:** Outcomes and complications. ED: emergency department. ^*^Significance set at p<0.05. ^**^Significance set at p<0.01. Student’s t-test was used to compare continuous variables, and chi-squared test was used to compare categorical variables, with alpha set to 0.05.

Variable	Overall (n=107)	Younger patients (age <70 years) (n=71)	Patients (age >70 years and older) (n=36)	p-value
Outcomes				
Intraoperative mortality, n (%)	6 (6)	0 (0)	6 (17)	<0.001**
30-Day mortality, n (%)	21 (20)	5 (7)	16 (44)	<0.001**
Postoperative length of stay, days (IQR)	9 (6-15)	9 (5-16)	8 (6-13)	0.397
Perioperative characteristics				
Transferred from outside hospital, n (%)	76 (71)	50 (70)	26 (72)	0.846
Time from presentation to diagnosis (minutes)	144 (64-260)	138 (62-235)	182 (103-312)	0.196
Time from presentation to case start (minutes)	405 (258-654)	385 (255-601)	433 (284-778)	0.020*
Time from diagnosis to case start (minutes)	242 (173-356)	234 (170-351)	262 (201-1085)	0.006**
Case length (minutes)	278 (239-356)	288 (239-350)	263 (224-346)	0.298
Cardiopulmonary bypass time (minutes)	143 (122-190)	143 (122-190)	139 (118-154)	0.472
Circulatory arrest time (minutes)	22 (18-28)	20 (17-25)	24 (20-28)	0.107
Aortic cross-clamp time (minutes)	92 (75-125)	92 (74-128)	80 (73-92)	0.449
Postoperative complications				
Postoperative bleeding requiring intervention, n (%)	30 (28)	22 (31)	8 (22)	0.584
Postoperative cerebrovascular accident, n (%)	21 (20)	14 (20)	7 (19)	0.209
Postoperative atrial fibrillation, n (%)	27 (25)	19 (27)	8 (22)	0.344
Postoperative pericardial window, n (%)	6 (6)	4 (6)	2 (6)	0.545
Postoperative thoracentesis, n (%)	25 (23)	20 (28)	5 (14)	0.647
Surgery-related ED visit, n (%)	43 (40)	32 (45)	11 (31)	0.610

**Figure 2:**
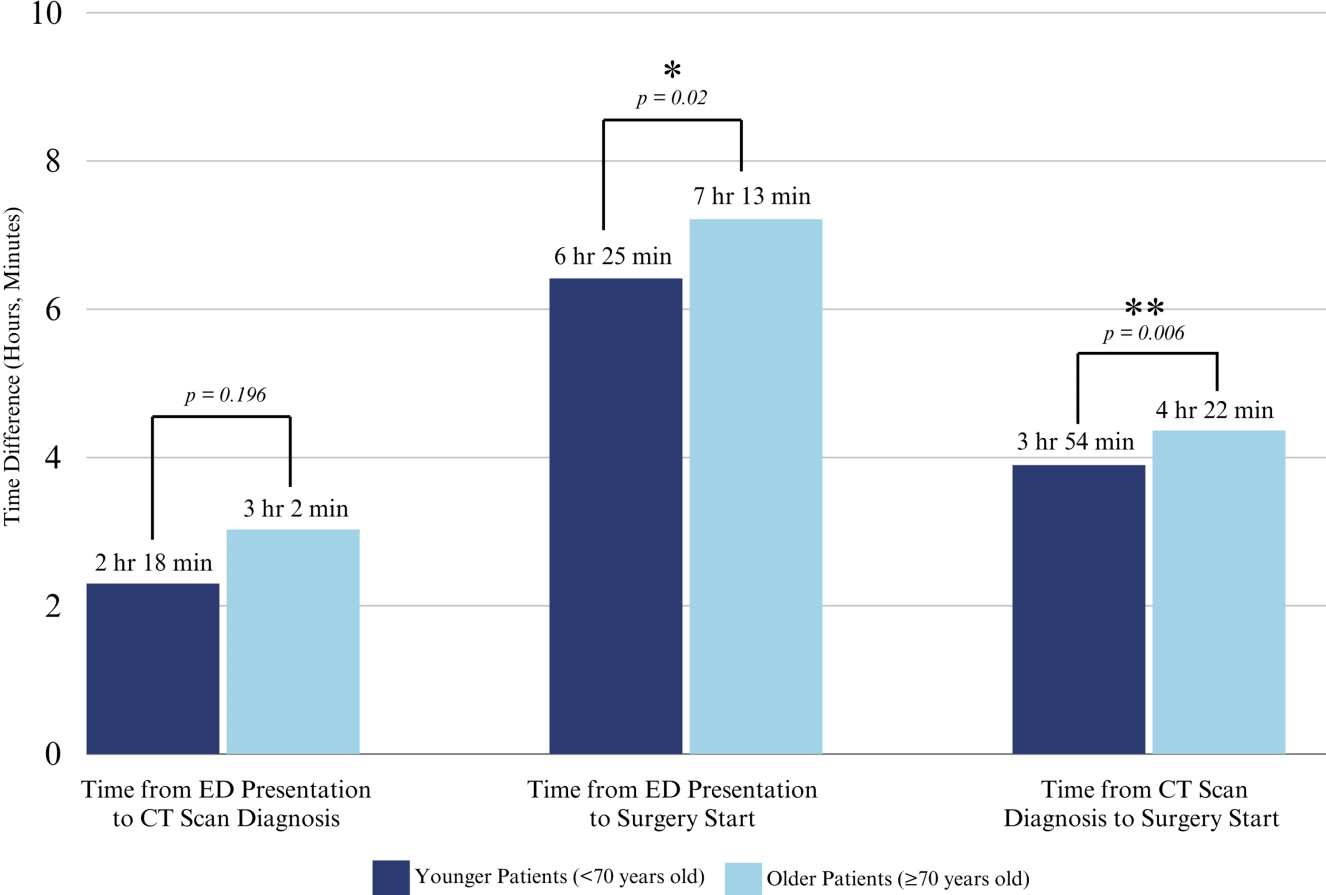
Time difference per group. CT: computed tomography; ED: emergency department.

Our analysis of the perioperative characteristics found no differences in the rate of transfers from an outside or community hospital to a tertiary care center (70% (n=71) vs. 72% (n=36), p=0.846). Interestingly, with this lack of difference in transfer rates, we observed longer times from presentation in the ED to the start of surgery for older patients compared to younger patients (7 hours and 13 minutes vs. 6 hours 25 minutes; p=0.02) and for time of diagnosis with CT scan to the start of surgery (4 hours 22 minutes vs. 3 hours 54 minutes (p=0.006). There were no differences in time from presentation in the ED to the diagnosis with CT scan when comparing older patients to younger patients (3 hours and 2 minutes vs. 2 hours and 18 minutes, p=0.196) (Figure 2; Table 2). Regarding other perioperative characteristics, there were no differences in case length, cardiopulmonary bypass time, circulatory arrest time, and aortic cross-clamp time (Table 2).

Our findings regarding secondary outcomes indicate that there were no differences in the rates of postoperative complications such as bleeding requiring intervention, cerebrovascular accident, atrial fibrillation, pericardial effusion requiring a pericardial window, pleural effusion requiring thoracentesis, and surgery-related ED visits (Table 2).

## Discussion

Our results indicate that both younger and older patients have a comparable amount of time elapsed from time of ED presentation to time of CT scan diagnosis. This observation is consistent with prior studies that have shown overall improved rates of prompt AAS diagnosis in the past few decades, likely due to the improvement and utilization of CT technology [2,6]. Although age did not impact diagnosis times, there is always room for improvement to minimize the time spent in the ED for every patient. Additionally, given that AAS may present with non-specific symptoms, there should be a low threshold to initiate prompt CT imaging for these patients when there is clinical suspicion. Imaging is not only useful in the detection and diagnosis of AAS but also in the surgical planning of aortic arch repair, for instance by providing a rapid picture of the dissection or by elucidating specific visceral branch involvement [11].

With no difference demonstrated in diagnosis time, our results showed that older patients experienced a significantly longer time from ED presentation to the start of surgery. This difference of 48 minutes is not only statistically significant but also clinically significant, given the established incremental increase in mortality with every hour that passes from the time of symptom onset [2-5]. Furthermore, we specifically found a significant increase in the time elapsed from CT scan diagnosis to case start for older patients; this finding demonstrates that a delay in care could occur after a patient is diagnosed with AAS. This delay may be a result of prolonged clinical decision-making due to the high morbidity and mortality associated with aortic arch repairs in older patients. While discussions regarding care should be sensitive and cognizant of the inherent risks of surgical management, they should not significantly impede or delay clinical decision-making for such a high-acuity disease process.

Some experts have hypothesized that the general hesitancy to operate on older patients results from a tendency toward risk aversion, and they further posit that there is often a lowered threshold set for determining the futility of care for older patients with AAS [12]. The literature on performing aortic surgery in the elderly is divided. Some papers have found that the decision to perform aortic surgery in the elderly should be approached with greater caution due to the associated high rates of preoperative comorbidity [13]. Other studies have found that older patients may actually demonstrate better outcomes than younger patients. A study by Tang et al. compared outcomes between 21 octogenarians with 80 concurrent patients aged less than 80 years undergoing acute type A aortic dissection repair [15]. This study found that while younger and older patients had comparable preoperative characteristics, octogenarians demonstrated favorable outcomes and improved quality of life [14-15]. In our study, we found that the older patient population experienced worse rates of intraoperative and 30-day mortality; however, it is difficult to determine if these outcomes were impacted by a delay to surgery after diagnosis, given the similar rates of preoperative comorbidities. Further qualitative studies are warranted to query surgeons’ possible hesitancy and clinical reasoning for delaying or withholding surgical management of aortic syndromes in the older patient population.

With the introduction of less invasive endovascular interventions and improvements in surgical techniques in the last few decades, older patients should still be considered for these treatment modalities if they are appropriate candidates. If aortic arch repair is promptly determined not to be futile, age should not be the precluding factor that prevents surgical management. Our findings call for an improved and more standardized means of operative risk stratification for aortic surgeries that considers not only age but the overall clinical picture. Mehta et al. have suggested a simple stratification rule that considers certain high-risk factors in the elderly that increase the risk for in-hospital mortality; these factors include hypotension, branch vessel involvement, and periaortic hematoma [8]. Further studies should be conducted to determine if the implementation of these strategies is effective in decreasing decision-making time and, subsequently, the time from presentation to surgery for patients of all ages.

Limitations

Limitations of our study include its retrospective nature. Furthermore, given the high mortality rate that this pathology carries, these data may not capture patients who died before evaluation and surgical management. Our study is further limited by our inability to characterize preoperative decision-making discussions and the clinician-based reasons for delay in care.

## Conclusions

Our study demonstrates that septuagenarian and octogenarian patients experience delays from the time of original presentation to eventual surgical management. Patients aged 70 and older experienced delays from the time of presentation to the start of surgery and from the time of diagnosis to the start of surgery. Age should not delay an individual from receiving timely transfer to a tertiary center for a higher level of care to better assess the patient’s operative candidacy and determine appropriate treatment. Age should not also preclude an appropriate surgical candidate from receiving surgical management that could improve their outcome, prognosis, and quality of life. Our findings underscore the ongoing significance of clarifying the concept of futile care in older patients with aortic pathologies. Moreover, we emphasize that there is room for improvement in the management of elderly patients with AAS.
